# Complete chloroplast genome of *Paphiopedilum emersonii* (Orchidaceae)

**DOI:** 10.1080/23802359.2020.1827069

**Published:** 2020-10-07

**Authors:** Feng-Luan Tang, Li-Li Deng, Hui-Zhen Qin, Yan-Cai Shi

**Affiliations:** Guangxi Key Laboratory of Functional Phytochemicals Research and Utilization, Guangxi Institute of Botany, Guangxi Zhuang Autonomous Region and Chinese Academy of Sciences, Guilin, China

**Keywords:** *Paphiopedilum emersonii*, orchid, chloroplast genome, phylogenetic analysis

## Abstract

*Paphiopedilum emersonii* is an endemic terrestrial orchid in China. In this study, the chloroplast genome of *P. emersonii* was determined from BGISEQ-500 sequencing data. The total chloroplast genome was 162,590 bp in length, consisting of a large single-copy region (LSC, 87,852 bp), a small single-copy region (SSC, 870 bp), and two inverted repeat regions (IRA and IRB, 36,934 bp, each). The complete chloroplast genome contains 131 genes, including 81 protein-coding genes, 38 tRNA genes, and 8 rRNA genes. In addition, the phylogenetic analysis indicates that *P. emersonii* was sister to *Paphiopedilum micranthum.* The chloroplast genome will contribute to the research and conservation of *P. emersonii*.

*Paphiopedilum emersonii* belongs to the Subfam. Cypripedioideae of Orchidaceae (Chen and Stephan 2009). It is native to Guangxi and Guizhou of China (Averyanov et al. 2005). *Paphiopedilum emersonii* is famous for its specialization, large lip, and gorgeous flowers and colors, also known as Slipper Orchid, which has high ornamental value and is one of the most popular Orchidaceae plants in the world (Chen and Stephan 2009). *Paphiopedilum emersonii* has been widely cultivated all over the world, and there is registered in the Royal Horticultural Society (RHS; Zeng et al. 2010). However, due to excessive man-made collection, trade, habitat destruction, and other reasons for a long time, the wild resources of *P. emersonii* has decreased rapidly and even become extinct. All species of *Paphiopedilum* have been listed in Appendix I of the Convention on International Trade in Endangered species of Wild Fauna and Flora, and international trade is prohibited (Luo et al. 2003). Therefore, we report the complete chloroplast genome of *P. emersonii*, in order to better understand the relationship between *P. emersonii* and related genera, and contribute to the effective conservation strategy of *P. emersonii.*

In this study, the complete genomic DNA was extracted from fresh leaves using a modified CTAB method (Doyle and Doyle 1987) and sequenced by the BGISEQ-500 platform. The samples were collected from Yachang Orchidaceae National Nature Reserve, Guangxi, China (24°44′N, 106°15′E) and the voucher specimen deposited at the Herbarium of Guangxi Institute of Botany, Guangxi Zhuang Autonomous Region and Chinese Academy of Sciences (specimen code *Paphiopedilum*_GX).

The clean reads were used to assemble the complete chloroplast genome by the GetOrganelle pipe-line (Jin et al. 2018), with the chloroplast genome of *Paphiopedilum micranthum* (MN_535014) as the reference sequences. The assembled chloroplast genome was annotated using the Geneious R11.15 (Kearse et al. 2012). The physical map of the chloroplast genome was generated using the online tool OGDRAW (Lohse et al. 2013). Finally, we obtained a complete chloroplast genome of *P. emersonii* and submitted to GenBank with accession number MT648789.

The total chloroplast genome of *P. emersonii* was 162,590 bp in length, consisting of a large single-copy region (LSC, 87,852 bp), a small single-copy region (SSC, 870 bp), and two inverted repeat regions (IRA and IRB, 36,934 bp, each). The complete chloroplast genome contained 131 genes, including 81 protein-coding genes, 38 transfer RNA (tRNA) genes, and 8 ribosomal RNA (rRNA) genes. The GC content of the chloroplast genome of *P. emersonii* was 35.6%.

To investigate the phylogenetic position of *P. emersonii*, a phylogenetic analysis was performed based on 13 complete chloroplast genome sequences of Orchidaceae. All sequences were aligned with the HomBlock pipeline (Bi et al. 2018) and subsequently checked manually in Bioedit v5.0.9 (Hall 1999). Then, the phylogenetic tree constructed by RAxML (Stamatakis 2014) with 1000 ultrafast bootstrap (UFBoot) replicates (Minh et al. 2013; Chernomor et al. 2016). The results showed that *P. emersonii* was sister to *P. micranthum* with 100% bootstrap support (Figure 1).

**Figure 1.**
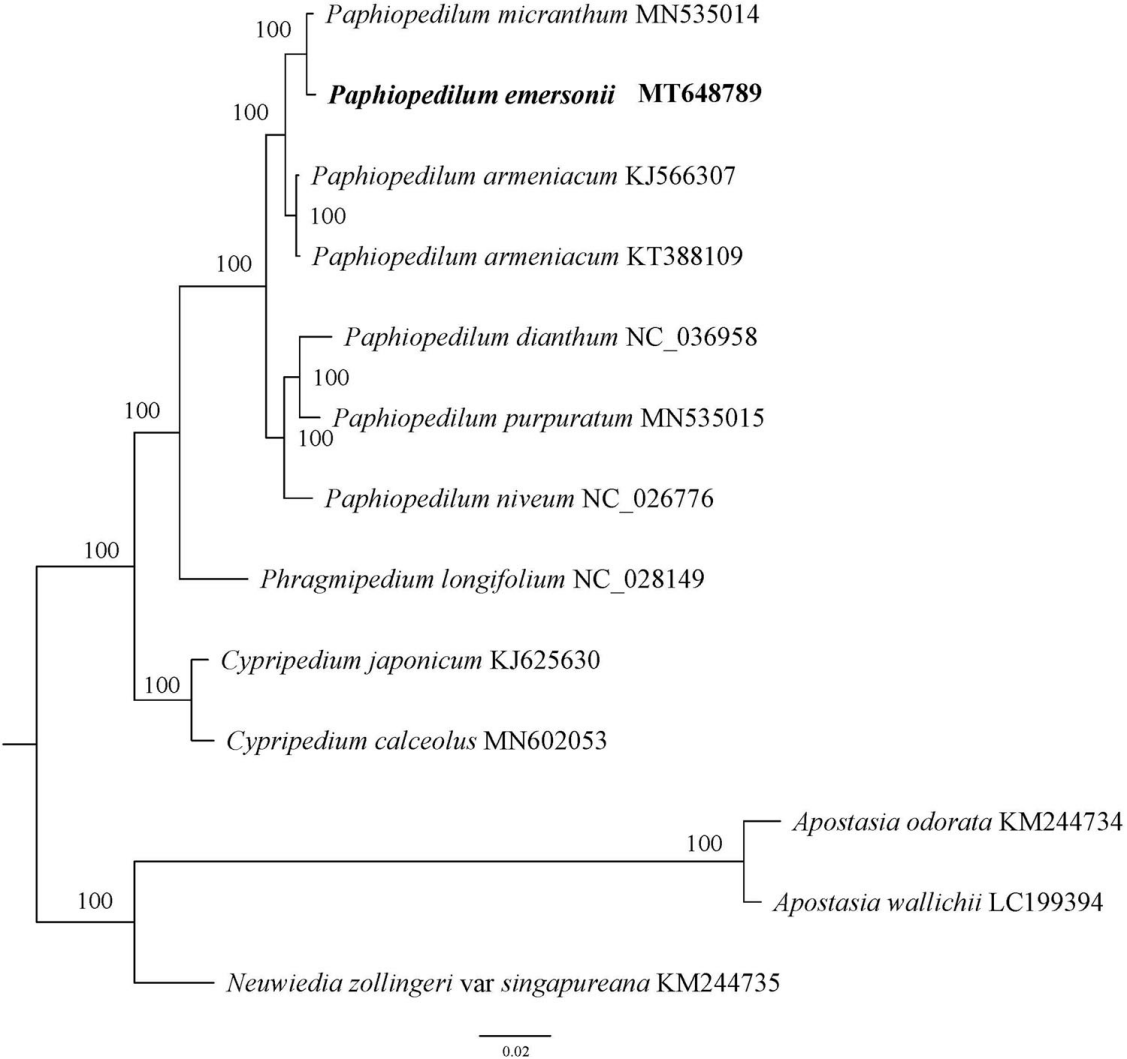
A phylogenetic tree constructed based on 13 complete chloroplast genome sequences of Orchidaceae. Bootstrap support is indicated for each branch.

## Data Availability

Data openly available in a public repository that does not issue DOIs. The data that support the findings of this study are openly available in the National Center for Biotechnology Information (https://www.ncbi.nlm.nih.gov/), reference number (MT648789).
